# Effects of Size and Geographical Origin on Atlantic salmon, *Salmo salar*, Mucin *O*-Glycan Repertoire*[Fn FN2]

**DOI:** 10.1074/mcp.RA119.001319

**Published:** 2019-03-28

**Authors:** John Benktander, Vignesh Venkatakrishnan, János T. Padra, Henrik Sundh, Kristina Sundell, Abarna V. M. Murugan, Ben Maynard, Sara K. Lindén

**Affiliations:** From the ‡Department of Medical Biochemistry and Cell Biology, Institute of Biomedicine, Sahlgrenska Academy, University of Gothenburg, Box 440, Medicinaregatan 9A, 405 30 Gothenburg, Sweden;; §Department of Biological and Environmental Sciences, University of Gothenburg, Sweden;; ¶The Commonwealth Scientific and Industrial Research Organisation, Hobart, Australia

**Keywords:** Glycomics, Mass Spectrometry, Tandem Mass Spectrometry, Glycoproteins*, Glycosylation, Glycoprotein Structure*, Mucins, Mucus, O-glycan

## Abstract

Diseases cause ethical concerns and economic losses in the Salmonid industry. The mucus layer comprised of highly *O*-glycosylated mucins is the first contact between pathogens and fish. Mucin glycans govern pathogen adhesion, growth and virulence. The Atlantic salmon *O*-glycome from a single location has been characterized and the interindividual variation was low. Because interindividual variation is considered a population-based defense, hindering the entire population from being wiped out by a single infection, low interindividual variation among Atlantic salmon may be a concern. Here, we analyzed the *O*-glycome of 25 Atlantic salmon from six cohorts grown under various conditions from Sweden, Norway and Australia (Tasmania) using mass spectrometry. This expanded the known Atlantic salmon *O*-glycome by 60% to 169 identified structures. The mucin *O*-glycosylation was relatively stable over time within a geographical region, but the size of the fish affected skin mucin glycosylation. The skin mucin glycan repertoires from Swedish and Norwegian Atlantic salmon populations were closely related compared with Tasmanian ones, regardless of size and salinity, with differences in glycan size and composition. The internal mucin glycan repertoire also clustered based on geographical origin and into pyloric cecal and distal intestinal groups, regardless of cohort and fish size. Fucosylated structures were more abundant in Tasmanian pyloric caeca and distal intestine mucins compared with Swedish ones. Overall, Tasmanian Atlantic salmon mucins have more *O*-glycan structures in skin but less in the gastrointestinal tract compared with Swedish fish. Low interindividual variation was confirmed within each cohort. The results can serve as a library for identifying structures of importance for host-pathogen interactions, understanding population differences of salmon mucin glycosylation in resistance to diseases and during breeding and selection of strains. The results could make it possible to predict potential vulnerabilities to diseases and suggest that inter-region breeding may increase the glycan diversity.

## Abstract (precis)

Mucin glycans govern pathogen adhesion, growth and virulence. We analyzed the *O*-glycome from six Atlantic salmon cohorts grown under various conditions from Sweden, Norway and Australia using LC-MS. The low interindividual variation identified may be a concern because interindividual variation is considered a population based defense against infection. The 169 identified structures represent a library for identifying structures important for host-pathogen interactions, understanding population differences of salmon mucin glycosylation in resistance to diseases and during breeding and selection of strains.

## Graphical Abstract

**Figure:**
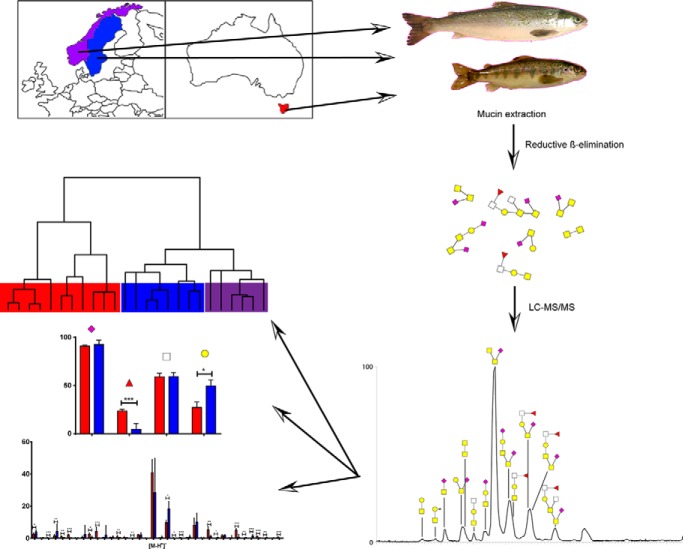

**Highlights**

- Atlantic salmon *O*-glycome expanded to 169 structures in three epithelia.
- Low interindividual variation amongst all populations and geographical regions.
- Small variations in glycosylation between geographical locations and fish size.
- Prominent fucosylation in gastrointestinal mucins from Tasmanian fish.

The ray-finned fish Atlantic salmon (*Salmo salar*) originating from the northern Atlantic Ocean is widely used in commercial aquaculture. Fish, including salmon, are a rich source of high-quality proteins, n-3 fatty acids, vitamin D, iodine, selenium, and antioxidants. The production of salmonids, mainly Atlantic salmon and rainbow trout, for human consumption is steadily growing. In aquaculture worldwide, disease outbreaks are a major limiting factor for the sustainable, ethical and economic development of the industry (1). Even though improved fish health management is one of the most important factors behind the growth of the salmonid industry in the two major salmon producing countries Scotland and Norway, stock losses of up to 20% during production are reported (2, 3).

A mucus layer covering the epithelial cells protects all surfaces of the fish exposed to the environment, including skin, gills and intestine. The main components of this barrier are the mucin glycoproteins (4, 5), which can have 50–90% of their weight composed of carbohydrates (6). These carbohydrates are mostly in the form of *O*-glycans, where the carbohydrate chain is linked to a *N*-acetylgalactosamine on serine or threonine of the protein backbone (7). The *O*-glycans can protect against pathogens by adhering to the bacteria, thereby preventing them from interacting with the epithelial cells, but some bacteria can also use them as an energy source (8, 9). The pathogen *Aeromonas salmonicida* binds to sialic acids on salmon *O*-glycans and uses uncapped *N*-Acetylglucosamine (GlcNAc)^1^ as an energy source (10, 11). In addition, viral pathogens have been shown to induce down-regulation of mucin mRNA in the gills and gut of common carp, increasing the risk of secondary infections (12).

The Atlantic salmon mucin *O*-glycans have previously been studied by Jin C *et al.* (13). The *O*-glycans were identified as short (2–6 monosaccharides) glycans of core type 1, 2, 3 and 5 in the skin whereas larger glycans (2–12 monosaccharides) with core type 1, 2 and 5 were detected in the intestinal tract. NeuAc was found in mucins from all epithelial sites whereas NeuGc and Kdn structures were only detected on skin mucins. Although clear glycosylation differences were found between epithelial sites, very low interindividual variation in the *O*-glycome was observed in this cohort (13). This is in sharp contrast to the large interindividual variation in the *O*-glycome among humans, which is a population-based defense against infection, preventing entire populations from becoming wiped out by a single infection. Thus, the low interindividual variation in Atlantic salmon may be of concern. Currently, it is not known whether this situation is general for Atlantic salmon or population-specific for a single geographical location. Decreased interindividual variation in response to inflammation has been demonstrated among mucin *O*-glycans from Artic charr (*Salvelinus alpinus*) (14).

Human gastric mucin *O*-glycans display a multitude of glycans with a large interindividual variation, where histo-blood group antigens are responsible for a considerable part of the diversity (15). Little is known about the natural variation of carbohydrate species and blood groups expressed in fish. A few studies from the 1960s found blood group systems in skipjack tuna (*Katsuwonus pelamis*) called the “Y system” with 15 phenotypes (16), rainbow trout (*Oncorhynchus mykiss*) with three phenotypes in a 2 allele system and brown trout (*Salmo trutta*) with a 4-phenotype system during maturity and a 2-phenotype system for younger fish (17). These studies found different immunogenic reactions but did not conclude whether these blood groups are composed of carbohydrates or proteins.

The aims of this study were to (1) expand the known Atlantic salmon *O*-glycome to cover more individuals from populations with differing sizes and life stages, as well as from different geographical regions and environmental conditions, (2) investigate if Atlantic salmon glycan interindividual variation is low in general, (3) identify if *O*-glycosylation is stable with time, fish size and environmental conditions, and (4) to identify potential geographic differences in *O*-glycan repertoire and interindividual diversity. In order to answer these questions, we also compared mucin preparation methods to achieve faster results with smaller sample quantities.

## EXPERIMENTAL PROCEDURES

### 

#### 

##### Salmon Growth and Sampling Procedure

Three freshwater cohorts of Atlantic salmon from Tasmania (Australia) were sampled. Cohort 1 (5 fish, 20 months old, 500–1000g, post-smolts retained in freshwater) and 2 (3 fish, 9 months old, 25–50g, parr) were from the Salmon Enterprises of Tasmania selective breeding program (SALTAS Pty Ltd, Wayatinah, Tasmania, Australia). The fish were reared in flow-through, 55,000 L tanks with freshwater from the Derwent River. They were sampled after capture by net and sacrificed by a sharp blow to the head. Cohort 1 were fed with 6 mm pellets and cohort 2 with 2 mm pellets (Skretting, Australia). Cohort 3 (3 fish, 25–30g, parr) were grown in freshwater basins using flow through water from the Dennison River (Snowy Range Hatchery, Dennison River, Australia) and fed 2 mm pellets (Skretting, Australia). Tasmanian Atlantic salmon are originally of wild type from River Philip (Nova Scotia, Canada) but have been bred in captivity in Tasmania since the 1960s and are considered a single strain.

The Swedish Atlantic salmon samples consisted of two cohorts (cohort 4: 2012, 5 fish, 250–300g, and cohort 5; 2015, 3 fish, 250–300g) that were kept under the same conditions: wild type fish were obtained from Långhults Lax AB (Långhult, Sweden) and kept in 500 L tanks at the Department of Biological and Environmental Sciences. The fish were held in recirculating freshwater, supplemented with 10% salt water (yielding a salinity of 2–3%), at a flow rate of 8.5 L/min. The fish were hand-fed *ad libitum* once daily with a commercial dry pellet (Nutra Olympic 3 mm; Skretting Averøy Ltd., Norway) and exposed to a simulated natural photoperiod. Fish were randomly netted, sedated in metomidate (12.5 mg/L), and killed with a blow to the head. The *O*-glycan profile of the 2012 cohort has previously been described in detail (13). The data from this 2012 cohort was re-examined for low abundance glycans and included in the current study.

Norwegian Atlantic salmon (cohort 6, 6 fish, mean weight ∼560 g) were obtained from Nofima Centre for Recirculation in Aquaculture (Sunndalsøra, Norway). Post-smolts were raised in a flow through system (FTS), simulating floating semi-closed containment systems in sea at full salinity.

Tissues were soaked with 10 mm sodium dihydrogen phosphate containing 0.1 mm phenylmethanesulphonyl fluoride (PMSF), pH 6.5 (sampling buffer) before collection. Skin mucus was sampled by gently scraping the entire fish skin surface using two glass microscope slides. Intestinal mucus was collected by cutting the dissected intestine along the mesenteric border, separating the proximal region from the distal at the ileorectal valve, and scraping off the mucus and mucosa with microscope slides. The pyloric caeca were removed using scissors and placed in liquid nitrogen for grinding using a mortar and pestle.

##### Mucin Extraction

Isolation of mucins: Mucins were extracted and isolated using isopycnic density gradient centrifugation as previously described (13).

Crude extraction: A crude extraction of mucins was made from the Tasmanian and Norwegian mucin scrapings by mixing each with two times the sample volume of extraction buffer (6 m guanidine hydrochloride (GuHCl), 5 mm EDTA, 10 mm sodium phosphate buffer (pH 6.5), 0.1 mm PMSF) and subjecting samples to four strokes with a loose pestle using a Dounce homogenizer. The resulting solution was transferred to a new tube together with another two sample volumes of extraction buffer used to rinse the homogenizer. Samples were incubated on a rocking board for 20 h at 4 °C, followed by centrifugation at 3900 × *g* for 80 min. Supernatants were collected and saved as crude mucin fractions at 4 °C.

##### O-glycan Analysis by LC-MS

Fish mucins (∼100 μg, from skin, pyloric caeca and distal intestine) were dot-blotted on PVDF membrane (Immobilon P membranes, Millipore, Billerica, MA) in order to remove the extraction buffer. The dots were visualized using Alcian blue (Sigma-Aldrich). Blue dots were cut out and subjected to reductive β-elimination. Briefly, the excised dots were incubated with 1 m NaBH_4_ and 50 mm NaOH for 16 h at 50 °C. 2 μl of glacial acetic acid was added to quench the reaction, and the samples were desalted with a AG50WX8 cation-exchange resin (Bio-Rad) in a reverse-phase μ-C18 ZipTip (Millipore). Eluates were dried in a SpeedVac concentrator and methanol was added and dried five times to evaporate borate in the sample. A side-effect of the reductive β-elimination reaction used is the likely removal of ester-bonds from *O*-acetylated sialic acids.

Released *O*-glycans were analyzed by liquid-chromatography-mass spectrometry (LC-MS) using a 10 cm × 250 μm i.d. column prepared in-house, containing 5 μm porous graphitized carbon (PGC) particles (Thermo Scientific, Waltham, MA). A linear LC gradient of 0–40% acetonitrile in 10 mm ammonium bicarbonate over 40 min, at a flow rate of ∼10 μl/min, was used to elute the *O*-glycans. The *O*-glycans were detected using an LTQ mass spectrometer (Thermo Scientific) in negative-ion mode with an electrospray voltage of 3.5 kV, capillary voltage of −33.0 V, and a capillary temperature of 300 °C. Compressed air was used as a sheath gas. In LC-MS, full scans were performed in the mass range of *m*/*z* 380–2000. MS/MS was performed at a normalized collisional energy of 35% with a minimal signal of 300 counts, isolation width of 2.0 *m*/*z*, and an activation time of 30 ms. The data were processed using Xcalibur software (version 2.0.7, Thermo Scientific). Glycans were manually annotated from their MS/MS spectra with the help of Glycoworkbench 2 software (18) and validated using the UniCarb-DB database (19) or a previous study by Jin *et.al* (13) if the structures were available. Some statistical assumptions were made during structural annotation: The monosaccharide at the reducing end was assumed to be GalNAcol, hexoses were assumed to be Gal residues and O-glycans with linear cores (core 1, 3, and 5) were differentiated from branched cores (core 2) based on the presence of (M–H)^−^−223 and (M–H)^−^−C_3_H_8_O_4_ (−108) in the MS/MS of structures with linear cores (13, 20). Structures were also identified by epitope specific fragmentation and biosynthetic pathways. For statistical analysis, the relative amount of glycan was calculated based on the precursor mass specific peak areas in the LC-MS chromatograms. The relative amount of each glycan was calculated as a percentage of the total area of all selected peaks and used for comparison. Proposed structures are represented using the Symbol Nomenclature for Glycomics (SNFG) (21).

##### Experimental Design and Statistical Rationale

Mucin *O*-glycans were characterized from 25 Atlantic salmon across six cohorts (*n* = 3–6/cohort). Skin *O*-glycans were characterized for all samples, whereas pyloric caeca and distal intestine *O*-glycans were characterized for 16 samples, because of low variation between cohorts in these regions. Because the interindividual variation within each cohort was very low, only biological replicates were analyzed (no technical replication), although a pig gastric mucin control was run on the mass spectrometer regularly to verify comparable MS results over time. Statistical analysis was performed using GraphPad Prism 7.0 (GraphPad Software Inc.). Non-parametric statistics were used because of the possibility of blood group structures. Mann-Whitney U-tests were used for comparing Tasmanian and Swedish salmon mucin *O*-glycans and structure categories. When comparing the Tasmanian, Swedish and Norwegian Atlantic salmon skin mucin samples, Kruskal-Wallis tests with Dunn′s multiple comparisons corrections were used. Spearman's rank correlation coefficients (ρ) were calculated comparing each individual sample using the relative abundance for each glycan structure. Only structures above 1% relative abundance were included to avoid differences because of variation in signal to noise ratio between datasets. Mann Whitney U-tests were used to compare differences between groups, *e.g.* if the distribution of ρ between cohorts was larger than within cohorts, or the distribution between cohorts differed more between certain geographical regions than others. The distribution of ρ was also used as a measure of diversity within groups. Hierarchical clustering was done with Cluster 3.0 (22) using Spearman rank correlation and centroid linkage. Cluster visualization was performed with Java TreeView (version 1.1.6r4) (23).

##### Ethics Approval

The experimental use of Swedish Atlantic salmon was approved by the Ethical Committee for Animal Experiments in Gothenburg, Sweden under license #46/2009 and #177/2013. The Norwegian fish were approved according to FOTS ID 7427. No ethics approval was needed for the Australian fish as they were opportunistically sampled in the course of routine commercial culling operations.

## RESULTS

### 

#### 

##### Crude and Purified Mucin Extracts Yield Similar O-glycan Profiles Using LC-MS

Previously, we have analyzed *O*-glycans from mucins purified by density gradient centrifugation (13). This method is time consuming and requires a large sample volume. Therefore, we compared *O*-glycan profiles from a crude extraction method with those from mucins isolated by density gradient centrifugation. Analyses of two Swedish Atlantic salmon proximal intestinal samples from cohort 5 revealed only minor differences, of a similar magnitude to those observed among technical replicates within extraction methods (supplemental Fig. S1). Therefore, crude extraction of mucins was used for the Tasmanian and Norwegian Atlantic salmon samples.

##### The Mucin O-glycosylation Was Relatively Stable Over Time Within One Geographical Region

In our previous study of the Atlantic salmon mucin *O*-glycome, we identified glycosylation differences between epithelial sites, however, we were surprised by the low interindividual variation in this cohort (13). To ensure that the results were consistent over time (*e.g.* that we had not accidentally analyzed five siblings), we investigated samples from another three Atlantic salmon of the same size (parr, ∼250g), from the same river by LC-MS. We compared *O*-glycans from mucins purified using density gradient centrifugation from the original cohort sampled in 2012 (cohort 4, *n* = 5, (13)) with fish sampled in 2015 (cohort 5, *n* = 3). Overall, if not counting structures with abundances near the detection limit, there were no structures present in the 2015 cohort that were absent in the 2012 cohort. However, we detected a few differences in relative abundance of certain structures: among skin mucin glycans, the only statistically significant difference was observed at *m/z* 878.3 (Fuc-HexNAc-(NeuGcα2–6)GalNAcol), with a slightly higher abundance in the 2015 cohort (supplemental Fig. S2*A*). In the pyloric caeca, the relative abundance of the short structures at *m/z* 384.2 (Galβ1–3GalNAcol), 425.2 (GalNAcα1–3GalNAcol), 587.2 (Galβ1–3(GlcNAcβ1–6)GalNAcol), 675.3 (Galβ1–3(NeuAcα2–6)GalNAcol), 749.3 (Galβ1–3(Galβ1-3GlcNAcβ1–6)GalNAcol), 790.3 (Gal-(HexNAc-)GalNAcα1–3GalNAcol) and 878.3 (HexNAc-Galβ1–3(NeuAcα2–6)GalNAcol) was lower, whereas the structure at *m/z* 716.3 (GalNAcα1–3(NeuAcα2–6)GalNAcol) was higher in the 2015 cohort than in the 2012 cohort (supplemental Fig. S2*B*). By MS^3^ on the ^0,4^A-fragment of the *m/z* 790 parent ion, the branching HexNAc were determined as a β1–6 linked GlcNAc because of the presence of *m/z* 99.9, 112.0, and 142.0 at similar intensities, and the lack of *m/z* 184 and 196 fragments (24) (supplemental Fig. S3). In the distal intestine, some subtle differences were observed, with a lower relative abundance of glycans at *m/z* 675.2 (Galβ1–3(NeuAcα2–6)GalNAcol), 878.3 (NeuAcα2–3HexNAc-Galβ1–3GalNAcol) and 1260.5 (Hex+Galβ1–3(Fuc-GlcNAc-Gal-GlcNAcβ1–6)GalNAcol), whereas a higher abundance was detected for *m/z* 1575.5 (NeuAcα2–3HexNAc-Gal-(HexNAcβ1–6)GalNAcα1–3(NeuAcα2–6)GalNAcol) when comparing the 2015 cohort to the 2012 cohort (supplemental Fig. S2*C*). The Spearman's rank correlation coefficients for glycan relative abundances between the two time points were 0.7, 0.6, and 0.7 for skin, pyloric caeca and distal intestine, respectively, with statistically significant differences between cohorts for pyloric caeca and distal intestine (Table I).

**Table I:** Correlation of mucin O-glycan abundances from fish at different time points of sampling and age

	2012, cohort 4 Median ρ (IQR)	2012 vs 2015, cohort 4 vs 5 Median ρ (IQR)	P cohort 4 vs 5	∼750 g, cohort 1 Median ρ (IQR)	∼750 g vs ∼25 g, cohort 1 vs 2 and 3 Median ρ (IQR)	P cohort 1 vs 2 and 3
Skin	0.77 (0.72–0.93)	0.74 (0.69–0.84)	0.22	0.73 (0.56–0.88)	0.43 (0.33–0.57) and 0.36 (0.21–0.49)	<0.001
Pylori caeca	0.81 (0.59–0.88)	0.61 (0.58–0.66)	<0.05	0.87 (0.78–0.91)	0.91 (0.87–0.93)	0.13
Distal intestine	0.81 (0.79–0.85)	0.70 (0.64–0.73)	<0.0001	0.90 (0.87–0.93)	0.83 (0.79–0.85)	<0.001

Left: Comparison of Spearman's rank correlation coefficients (ρ) from within the 2012 cohort and between 2012 and 2015 cohorts (4 and 5). Right: Comparison of ρ from within the ∼750 g cohort (1) and between the ∼750 g cohort and the two ∼25 g cohorts (2 and 3). Because of differences in signal amplitude between samples, only structures with a relative abundance above 1% in at least one sample were correlated to minimize the risk of detecting differences caused by MS detection threshold. Statistics: The values show the median with the interquartile range in parentheses, with *p* values calculated using the Mann-Whitney U-test. This analysis is presented to demonstrate the inter- and intracohort variations, i.e. not to point to whether associations are statistically significant.

##### Fish Size Affects Skin Mucin Glycosylation

To investigate if fish size or culture facility/water source affect the *O*-glycan repertoire, *O*-glycans from skin, pyloric caeca and distal intestine were compared from three Tasmanian cohorts: cohort 1 (∼750 g, SALTAS/Derwent River), 2 (∼25 g, SALTAS/Derwent River) and 3 (∼25 g, Snowy Range Hatchery/Dennison River). The three cohorts had relatively similar *O*-glycosylation, with no clear differences detected between the two ∼25 g cohorts, although some differences were found comparing ∼25 g cohorts with the ∼750 g cohort: The ∼750 g cohort consistently expressed Gal-GalNAcα1–3GalNAcol (*m*/*z* 587.2) in the skin, which was absent among the ∼25 g cohorts, whereas the ∼25 g fish did not consistently display any major *O*-glycan structures not also present in the ∼750 g cohort. The differences were most obvious in the skin mucins (median ρ skin 0.4, pyloric caeca 0.9, distal intestine 0.8, Table I) where the *O*-glycan MS-ions at *m/z* 587.2 (Gal-GalNAcα1–3GalNAcol), 952.3 (Gal-(Gal-HexNAcβ1–6)GalNAcα1–3GalNAcol) and 1301.4 (Fuc-HexNAc-Gal-(Gal-HexNAcβ1–6)GalNAcα1–3GalNAcol) exhibited a higher relative abundance in cohort 1 compared with cohort 2 (supplemental Fig. S2*D*). Comparing cohort 1 and 3 significant differences were found in the relative abundances of *m/z* 587.2 (Gal-GalNAcα1–3GalNAcol), 593.2 (SO_3_+NeuAcα2–6GalNAcol), 675.2 (NeuAcα2–3Galβ1–3GalNAcol), 966.2 (NeuAcα2–3Galβ1–3(NeuAcα2–6)GalNAcol), and 1342.6 (HexNAc-(Fuc-)HexNAc-Gal-(HexNAc1–6)GalNAcα1–3GalNAcol). In pyloric caeca and distal intestine mucins the differences were more subtle with *m/z* 513.2 (NeuAcα2–6GalNAcol), 1081.4 (Gal-(HexNAc1–6)GalNAcα1–3(NeuAcα2–6)GalNAcol), and 1227.4 (Fuc-HexNAc-Gal-GalNAcα1–3(NeuAcα2–6)GalNAcol) slightly higher in the pyloric caeca from the ∼25 g fish, whereas only *m/z* 1007.2 (GalNAcα1–3(NeuAcα2–8NeuAcα2–6)GalNAcol) was higher in the distal intestine (supplemental Fig. S2*E*–S2*F*). Thus, Atlantic salmon size influences skin *O*-glycosylation, but has less effect on glycans in the intestinal tract.

##### Swedish and Norwegian Skin Mucin Glycan Repertoires Were More Closely Related to Each Other Compared With Tasmanian Ones, Regardless of Size and Salinity

**Fig. 1.**
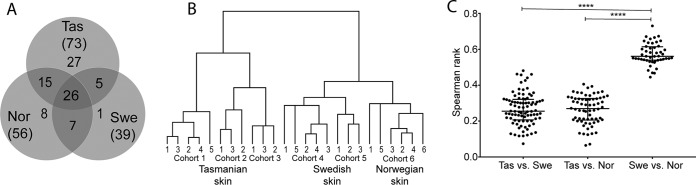
**Relation between Atlantic salmon skin mucin *O*-glycans from different geographical regions.** *A*, Venn diagram of *O*-glycans found among Tasmanian (Tas, *n* = 11: Cohort 1, 2 and 3), Swedish (Swe, *n* = 8: Cohort 4 and 5) and Norwegian (Nor, *n* = 6) salmon skin mucins. *B*, Tree view after hierarchal clustering of *O*-glycans from Atlantic salmon skin mucins. The hierarchical clustering was performed using Spearman rank correlations with centroid linkage. *C*, Spearman rank correlation coefficient r (ρ) values between individual skin mucin glycan profiles from the different locations. Significance was calculated using Kruskal-Wallis tests with Dunn′s multiple comparison corrections.

Atlantic salmon mucin *O*-glycans from Tasmania (cohort 1, *n* = 5; ∼750 g, 2 and 3, *n* = 3; ∼25 g, freshwater parr from two different facilities), Sweden (cohort 4, *n* = 5, and 5, *n* = 3, freshwater parr from the same facility, 3-years apart, ∼270 g) and Norway (cohort 6, *n* = 5, salt water parr, ∼560 g) were compared. This resulted in finding 73, 39 and 56 different *O*-glycan structures in Tasmanian, Swedish, and Norwegian salmon, respectively, with a size range of 2–9 monosaccharides (Fig. 1*A*). Twenty-six Atlantic salmon skin mucin structures were common to Tasmanian, Swedish and Norwegian samples, whereas 27, 1 and 8 structures were unique to these regions, respectively (Fig. 1*A*). Hierarchal clustering of the structures revealed that individual skin mucin samples were separated by their geographical origins (Fig. 1*B*). Spearman rank correlation showed a stronger relationship between the Swedish and Norwegian Atlantic salmon skin mucin *O*-glycans compared with the Tasmanian cohort *versus* any other (Fig. 1*C*, *p* < 0.0001). Within each geographical origin, the individuals clustered together according to their cohorts, with the Tasmanian ∼25 g cohorts forming a cluster separate from the ∼ 750 g cohort (Fig. 1*B*). This suggests that fish size and culture conditions affect mucin *O*-glycosylation, although geographical/strain differences have larger impact.

##### In Contrast to Norwegian and Swedish Atlantic Salmon Skin Mucin O-glycans, Tasmanian O-glycans Infrequently Contain NeuGc

**Fig. 2.**
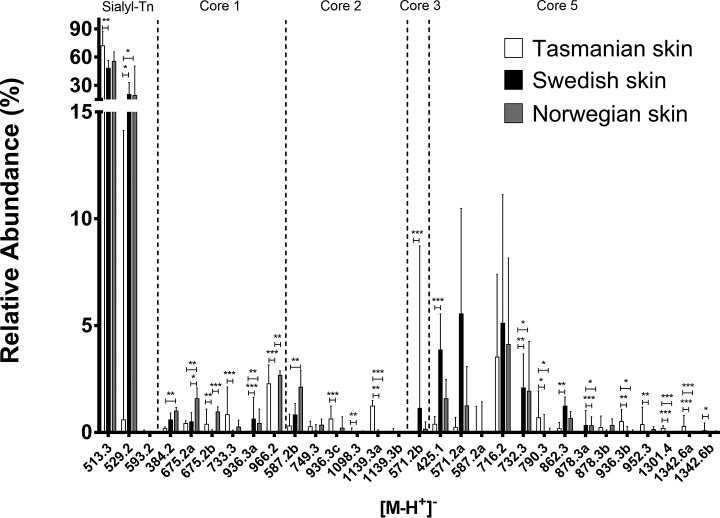
**Relative abundances of Atlantic salmon skin mucin *O*-glycans from different geographical regions.** Tasmanian (cohort 1, 2 and 3, *n* = 11), Swedish (cohort 4 and 5, *n* = 8) and Norwegian (cohort 6, *n* = 6) Atlantic salmon skin mucin *O*-glycans with at least one value above 1% were compared (for all *O*-glycans see supplemental Table S1). Error bars show the interquartile range. Significance was calculated using Kruskal-Wallis tests with Dunn′s multiple comparison corrections.

All skin mucins contained a high abundance of short sialylated structures regardless of cohort. The main glycan of the spectra was sialyl-Tn with 40–80% relative abundance (supplemental Fig. S4). Compared with Swedish skin mucins, many *O*-glycans were differently expressed in the Tasmanian Atlantic salmon: *e.g.* some of the non-sialylated core 5 structures such as *m/z* 790.3 (Gal1–3(GlcNAcβ1–6)GalNAcα1–3GalNAcol) and *m/z* 952.3 (Gal1–3(Gal-GlcNAcβ1–6)GalNAcα1–3GalNAcol) (Fig. 2 and supplemental Fig. S3). The only difference between Norwegian and Swedish salmon skin mucin *O*-glycans was a higher relative abundance of the moderately expressed sialylated and di-sialylated core 1 structures at 675.2a (RT∼10.1 min, Galβ1–3(NeuAcα2–6)GalNacol), 675.2b (RT∼11.6 min, NeuAcα2–3Galβ1–3GalNAcol), and 966.2 (NeuAcα2–3Galβ1–3(NeuAcα2–6)GalNacol) in the Norwegian samples (*p* < 0.05, Fig. 2).

##### Glycan Size and Monosaccharide Composition Differ Between Tasmanian and Scandinavian Skin Mucin O-glycans

**Fig. 3.**
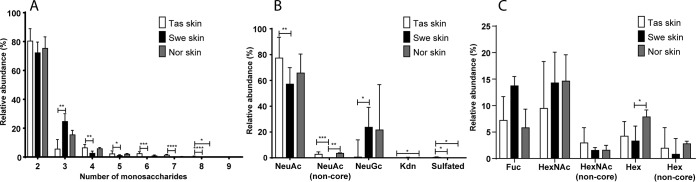
**Comparison of size and composition of *O*-glycans from Atlantic salmon skin mucins.** *A*, Size of the *O*-glycans (number of monosaccharides in the structures identified) on Tasmanian (Tas), Swedish (Swe) and Norwegian (Nor) skin mucins. *B*, Relative abundance of structures containing acidic monosaccharides. *C*, Relative abundance of structures containing neutral terminal monosaccharides. Non-core signifies exclusion of the structures with terminal glycans from the core structures or sialyl-Tn. The hexoses are most likely galactose, as terminal mannose and glucose to our knowledge have not been identified on *O*-glycans. Error bars show the interquartile range. Significance was calculated using Kruskal-Wallis tests with Dunn′s multiple comparison corrections.

The majority of structures from all cohorts were short; around 70–90% of the *O*-glycans were two residues and 5–25% were three residues long (Fig. 3*A*). Tasmanian skin glycans contained the highest abundance of large structures (6–9 monosaccharides, mean 4.2%, *p* < 0.0001) followed by Norwegian ones (mean 1.1%) whereas barely any large glycans could be identified among Swedish skin glycans (mean 0.1%, Fig. 3*A*). Approximately 80–90% of the structures from all cohorts were sialylated and the most common sialic acid was Neu5Ac (Fig. 3*B*). Structures containing Kdn and NeuGc were present on mucins from all fish of Scandinavian origin. In contrast, structures containing Kdn could only be found in the ∼25 g cohorts of Tasmanian origin (Fig. 3*B*). NeuGc was only found in one of the ∼750 g fish but was present in all the ∼25 g fish, albeit with less than 1% relative abundance (Fig. 3*B*). In contrast, NeuGc containing structures comprised more than 5% of the relative abundance of structures among Swedish and Norwegian skin mucin glycans (*p* < 0.05). Fucosylated structures were observed at an average ∼10% relative abundance, whereas terminal HexNAcs and Hex (Fig. 3*C*, non-core) were less abundant with averages lower than 4%. Including the terminal HexNAc and Hex found in the core structures yielded an average of ∼13% terminal HexNAcs, whereas the terminal Hex structures constituted on average 5 and 4% of Tasmanian and Swedish *O*-glycans, respectively. Terminal Hex structures were more abundant in the Norwegian cohort with an average 8% relative abundance.

##### The Internal Mucin Glycan Repertoire Cluster Into Pyloric Caeca and Distal Intestine Swedish and Tasmanian Populations, Regardless of Cohort and Size

**Fig. 4.**
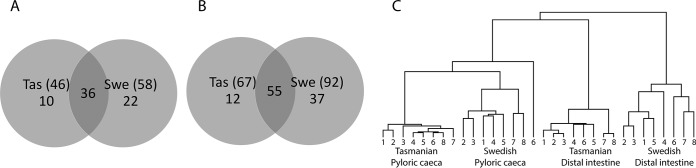
**Relation between Atlantic salmon pyloric caeca and distal intestine mucin *O*-glycans from different geographical regions.** *A*, Venn diagram of *O*-glycans found on pyloric caeca mucins from Tasmanian (Tas) and Swedish (Swe) Atlantic salmon. *B*, Venn diagram of *O*-glycans among distal intestine mucins. *C*, Tree view after hierarchal clustering of mucin *O*-glycans from pyloric caeca and distal intestine. The hierarchical clustering was performed using Spearman rank correlations with centroid linkage. Tasmanian 1–5: ∼750 g fish (cohort 1), Tasmanian 6–8: ∼25 g fish (cohort 2), Swedish fish 1–5: ∼270 g, sampled 2012 (cohort 4) (13), Swedish 6–8: ∼270 g, sampled 2015 (cohort 5).

Analysis of Atlantic salmon mucin *O*-glycans from Tasmania (cohort 1, *n* = 5, and 2, *n* = 3, freshwater parr; same origin, feed, and facility but differing in size ∼750 g *versus* ∼25 g) and Sweden (cohort 4, *n* = 5, and 5, *n* = 3, freshwater parr, same origin, feed, size, and facility but collected 3-years apart, ∼270 g), identified 46 structures from Tasmanian and 58 from Swedish pyloric caeca, of which 36 were common to both regions (Fig. 4*A*). In the distal intestine, 67 were identified among Tasmanian fish and 92 among Swedish ones, of which 55 were present in both geographical regions (Fig. 4*B*). Representative LC-MS chromatograms from both sources are shown in supplemental Fig. S4. Hierarchical clustering revealed four groups in the tree-view, according to the geographical origin and body location: For each body location, the ∼25 g and ∼750 g Tasmanian Atlantic salmon were similar (Fig. 4*C*). The small differences among the Tasmanian samples suggest that geographical location has more impact on *O*-glycan expression than fish size.

##### Increased Amounts of Fucosylated Structures in Tasmanian Pyloric Caeca and Distal Intestine Mucins Compared With Swedish Samples

**Fig. 5.**
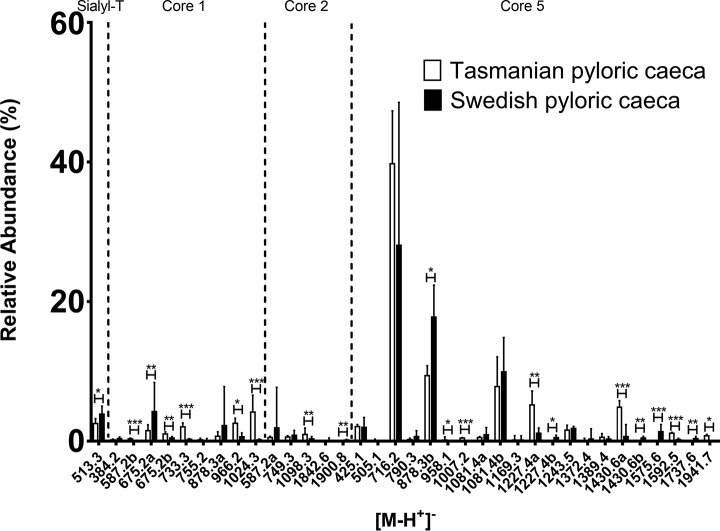
**Relative abundances of mucin *O*-glycans from Tasmanian and Swedish Atlantic salmon pyloric caeca.** Mucin *O*-glycans with at least one value above 1% were compared (for all *O*-glycans see supplemental Table S1). Diagram shows *O*-glycan structure masses sorted by core structure. Error bars show the interquartile range. Significance was calculated using Mann-Whitney U-tests (* *p* value <0.05, ** *p* value <0.01, *** *p* value <0.001).

**Fig. 6.**
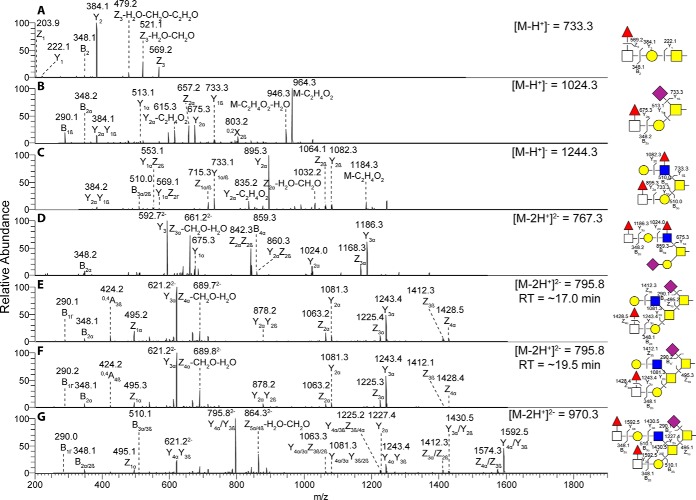
**Annotated MS/MS of fucosylated structures.** MS/MS of fucosylated structures with significantly higher relative abundance among pyloric caeca mucin *O*-glycans in Tasmanian compared with Swedish salmon. Symbols represents: *yellow circle*, Gal; *yellow square*, GalNAc; *blue square*, GlcNAc; *empty square*, HexNAc; *red triangle*, Fuc; *purple diamond*, NeuAc. Detailed assumptions related to linkage configuration and position, and validation of assigned structures can be found in materials and methods. Proposed structures are depicted using the Symbol Nomenclature for Glycomics (SNFG).

The major pyloric caeca *O*-glycans were composed of core 5 although some structures of intermediate expression were core 1 (Fig. 5). In addition, some low abundance glycans were of core 2. A low amount of sulfated structures were found but no structures with Kdn or NeuGc. Compared with Swedish pyloric caeca, several structures with terminal Fuc-HexNAc were more abundant in Tasmanian samples, including *m/z* 733.3 (Fuc-HexNAc-Galβ1–3GalNAcol), 1024.3 (Fuc-HexNAc-Galβ1–3(NeuAcα2–6)GalNAcol), 1244.3 (Fuc-HexNAc-Galβ1–3(Gal-(Fuc-)GlcNAcβ1–6)GalNAcol), 1535.5 (NeuAc-Galβ1–3(Fuc-HexNAc-Gal-(Fuc-)GlcNAcβ1–6)GalNAcol), 1592.5 (Gal-GlcNAcβ1–6(Fuc-HexNAc-Gal1–3)GalNAcα1–3(NeuAcα2–6)GalNAcol,RT ∼17.0 min), 1592.5 (Gal-GlcNAcβ1–6(Fuc-HexNAc-Gal1–3)GalNAcα1–3(NeuAcα2–6)GalNAcol, RT ∼19.5 min) and 1941.7 (Fuc-HexNAc-Gal-GlcNAcβ1–6(Fuc-HexNAc-Gal1–3)GalNAcα1–3(NeuAcα2–6)GalNAcol) (Fig. 5 and 6).

The fucosylated structures composed of core 1 and core 5 had the fucose linked to the terminal HexNAc, however core 2 *O*-glycans contained both terminal fucosylation of HexNAc as well as internal fucosylation of GlcNAc. The terminal fucosylation yielded some unusual fragments in MS/MS with a fragment from a loss of 212 u. It has been suggested that this fragment is because of a loss of terminal fucose linked to a HexNAc that will result in further loss of a H_2_O, forming a monoene from either C3 and C4 of the HexNAc and further inducing a dual elimination on the HexNAc to a diene, resulting in a loss of a Z-fucose fragment and H_2_O-CH_2_O (13).

**Fig. 7.**
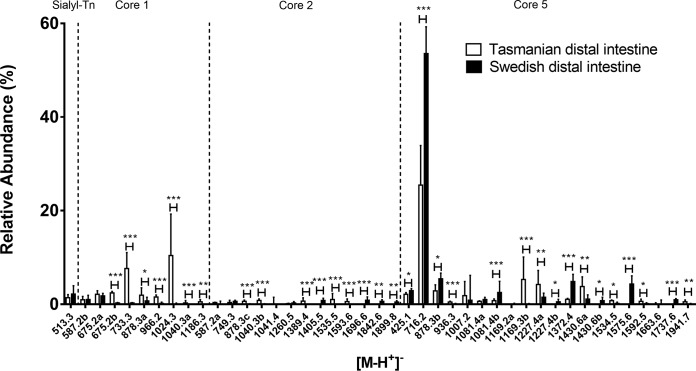
**Relative abundances of Atlantic salmon distal intestinal mucin *O*-glycans from Tasmania and Sweden.** Mucin *O*-glycans with at least one value above 1% were compared (for all *O*-glycans see supplemental Table S1). The diagram shows *O*-glycan structure masses sorted by core structure. Error bars show the interquartile range. Significance was calculated using Mann-Whitney U-tests (* *p* value <0.05, ** *p* value <0.01, *** *p* value <0.001).

**Fig. 8.**
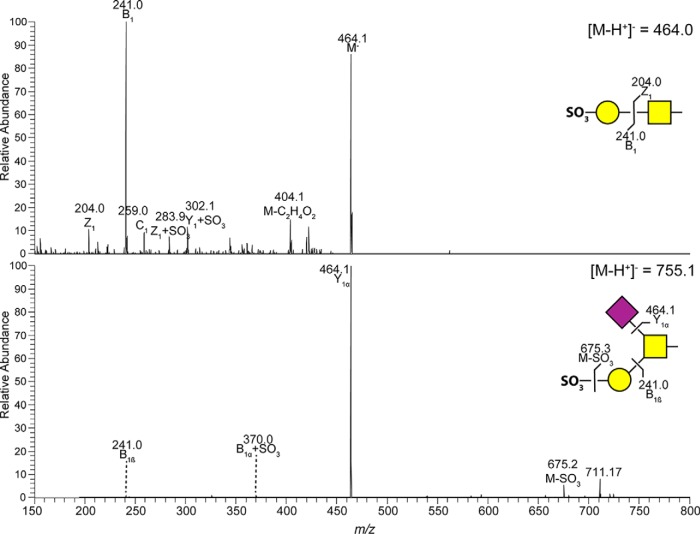
**MS/MS of sulfated *O*-glycans from Tasmanian Atlantic salmon distal intestinal mucins.** Some of the fragment ions occur because of sulfate migration (+SO_3_) (33). Proposed structures are illustrated using CFG symbol nomenclature with sulfate groups shown as SO_3_.

The most abundant *O*-glycans from Tasmanian Atlantic salmon distal intestine mucins were composed of core 5 (Fig. 7). In addition, some low abundance glycans were composed of core 1 and core 2. Trace amounts of sulfated and NeuGc containing structures were found. MS/MS of sulfated glycans found in distal intestine are shown in Fig. 8.

Similar to the pyloric caeca mucins, the Tasmanian distal intestine mucins displayed several fucosylated structures of higher relative abundance compared with Swedish samples *e.g. m/z* 733.3 (Fuc-HexNAc-Galβ1–3GalNAcol), 1024.3 (Fuc-HexNAc-Galβ1–3(NeuAcα2–6)GalNAcol), and 1430.6a (Fuc-HexNAc-Gal1–3(GlcNAcβ1–6)GalNAcα1–3(NeuAcα2-6)GalNAcol, RT 16.1 min). In contrast, some relatively abundant di-sialylated core 5 structures (*m/z* 1372.3 and 1575.5) in Swedish distal intestines exhibited a lower relative abundance in Tasmanian individuals. However, the shorter di-sialylated structure at *m/z* 1169.2c (NeuAcα2–3Gal-GalNAcα1–3(NeuAcα2–6)GalNAcol, RT 13.2 min) showed an increased relative abundance.

**Fig. 9.**
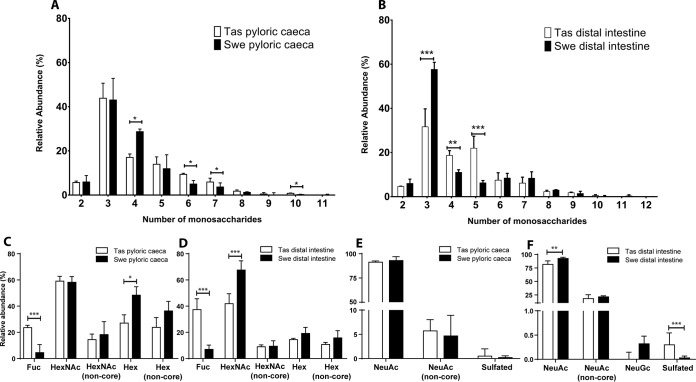
**Glycan size and terminal components on Tasmanian (Tas) and Swedish (Swe) mucins from the pyloric caeca and distal intestine.** *A–B*, Size of the *O*-glycans (number of monosaccharides in the structures identified) pyloric caeca (*A*) and distal intestine (*B*) mucins. *C* and *D*, Relative abundance of *O*-glycan structures with terminal neutral monosaccharides from the pyloric caeca and distal intestine mucins. “Non-core” signifies exclusion of structures with terminal monosaccharide in the core. The hexoses are most likely galactose, as terminal mannose and glucose to our knowledge have not been identified on *O*-glycans. *E* and *F*, Relative abundance of *O*-glycan structures with acidic constituents from pyloric caeca and distal intestine mucins. “Non-core” signifies exclusion of structures with a reducing end X-(NeuAcα2–6)GalNAc (Sialyl-Tn). Error bars show the interquartile range. Significance was calculated using Mann-Whitney tests. * *p* value <0.05, ** *p* value <0.01, *** *p* value <0.001.

In both geographical regions, the mucin *O*-glycans from the pyloric caeca and distal intestine were 2–12 residues long, whereof the majority were 3–5 residues (Fig. 9*A*). A higher proportion of large *O*-glycans (6+ residues, mean 18.17%, *p* < 0.05) were present among the Tasmanian pyloric caeca compared with Swedish samples (mean 11.59%), which was compensated for by a lower amount of 4-residue structures (Fig. 9*A*). In the distal intestine, the proportion of large *O*-glycans (6–12 residues) were similar between the geographical regions, however the proportion of glycans 4–5 residues long was higher among Tasmanian samples (*p* < 0.001), whereas the proportion of 3-residue structures was lower (*p* < 0.01). Furthermore, structures with a size of 11 and 12 monosaccharides were only detected in the Swedish cohort, albeit at very low abundance (Fig. 9*B*). Overall, large mucin *O*-glycan structures were more common among Tasmanian samples compared with Scandinavian samples, for both external and internal body locations.

The Tasmanian mucins had on average more than twice the amount of fucosylated structures in both pyloric caeca and distal intestine compared with Swedish mucins. Terminal Hex were higher in the Swedish pyloric caeca and HexNAc in the distal intestine, however no statistical significance were found when excluding terminal Hex and HexNAc found in the core structures (Fig. 9*C* and 9*D*). In the distal intestine the Tasmanian fish also had less NeuAc and a higher abundance of sulfated structures compared with Swedish samples (Fig. 9*E* and 9*F*).

##### Tasmanian Atlantic Salmon Mucins Have More O-glycan Structures in Skin but Less in the Gastrointestinal Tract Compared With Swedish Fish

**Fig. 10.**
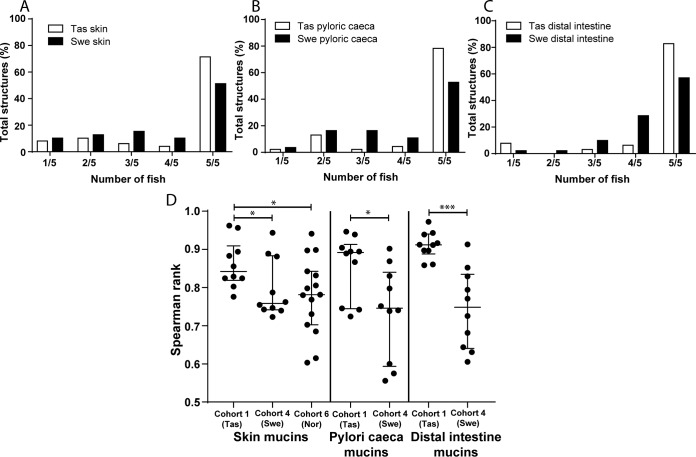
**O-glycan diversity and distribution among cohorts.** *A*, Distribution of skin mucin *O*-glycan structures among fish in Tasmanian (Tas, cohort 1) and Swedish (Swe, cohort 4) Atlantic salmon: bars represent the percentage of total glycan structures identified in only one fish (1/5) and up to 5 fish (5/5) in each group. *B*, Distribution of the pyloric caeca mucin *O*-glycan structures among fish in respective populations. *C*, Distribution of the distal intestine mucin *O*-glycan structures among fish in respective populations. *D*, Spearman′s rank correlations (ρ) of relative abundance of the glycan structures within cohorts were compared: skin (Tas-Swe: *p* = 0.0355, Tas-Nor: *p* = 0.0475, Swe-Nor: *p* = ns), pyloric caeca (*p* = 0.0355) and distal intestine (*p* = 0.0003). Significance was calculated using Mann-Whitney U-tests.

Among skin mucins, Swedish Atlantic salmon had the lowest number of glycans identified, though they had more glycan structures identified in the pyloric caeca and distal intestine (Fig. 1 and 4). Furthermore, tree-views from the hierarchal clustering (Fig. 1*B* and 4*C*) suggested that interindividual variation differed between geographical areas. The majority of the glycans were found in all Tasmanian fish (Fig. 10*A*–10*C*). To compare interindividual diversity within geographical locations, we only used samples from single cohorts to avoid introducing diversity because of culture/size differences. Cohort 1 (Tasmanian, *n* = 5) skin had 71% of structures in common whereas the pyloric caeca 78% and the distal intestine 83% (Fig 10*A*–10*C*). The corresponding numbers for cohort 4 (Swedish 2012, *n* = 5) were 51% of structures in common among skin glycans whereas 53% for pyloric caeca and 57% for distal intestine. The relative abundance of these common structures in the fish constituted on average 94% of the total *O*-glycans in skin, 97% in pyloric caeca and 99% in distal intestine for cohort 1. The lower percentage for skin mucins is because of one of the fish expressing NeuGc. The corresponding values in cohort 4 were 97% in skin, 90% in pyloric caeca and 95% in distal intestine. We analyzed the relation between the glycan profiles of each fish within cohorts and used the Spearman rank correlation coefficient as a measure of diversity within each cohort and mucin source. Results (Fig 10*D*) showed that the Tasmanian fish had a lower diversity among skin glycans than the Swedish and Norwegian fish. Similarly, the glycan diversity in the pyloric caeca and distal intestine was also lower among Tasmanian than Swedish fish (Fig. 10*D*).

## DISCUSSION

In the present study, we expanded the known Atlantic salmon *O*-glycome by 60% to 169 identified structures by analyzing skin, pyloric caeca and distal intestine mucins from fish of different sizes, life stages and geographical locations from a range of culture conditions. Mucin *O*-glycosylation was relatively stable over time within a geographical region. Fish size affected skin mucin glycosylation but had less impact on glycosylation of the gastro-intestinal tract. The skin mucin glycan repertoires of Swedish and Norwegian Atlantic salmon were more closely related compared with the Tasmanian population, regardless of size and salinity. The gastro-intestinal tract mucin glycan repertoire also clustered based on geographical origin and into pyloric caeca and distal intestine groups, regardless of cohort and fish size. Overall, Tasmanian Atlantic salmon mucins carried a larger repertoire of *O*-glycan structures in skin but a smaller repertoire in the gastrointestinal tract compared with Swedish fish. Low interindividual variation was confirmed within each cohort, and even more so among Tasmanian than Scandinavian fish.

Mucin isolation and purification using isopycnic density gradient centrifugation is time consuming and requires large tissue samples. To decrease the sample size needed and increase processing efficiency to enable a greater number of individual fish samples to be analyzed during this study, we compared the density gradient method with crude extraction, as well as a third method that included a composite gel separation step (not shown). The rational was that the risk of contaminating non-mucin glycans should be lower from the density gradient centrifugation method and the gel separation method, however only minor differences (of similar magnitude as those expected between technical replicates) were detected across methods. This suggests that the amount of *O*-glycans from extraneous sources are negligible in the mucus. Thus, for the current study we found that crude extraction was a reliable method, which allowed the processing of smaller samples.

With the recent advancement in the field of mass spectrometry, structural elucidation of mucin glycans has become more accurate. Although structures must be annotated manually because of microheterogeneity, a reliable database of mucin glycan structures of potential importance for the study of host/pathogen interactions offers many advantages over faster analyses determining only monosaccharide composition. By increasing the number of Atlantic salmon analyzed 5-fold during the present study, we increased the *O*-glycome for this species by 60%. Pathogens such as *A. salmonicida* bind to *N*-acetylneuraminic acid on Atlantic salmon mucins and use mucin-associated *N*-acetylglucosamine as a nutrient source (10, 11), fish-pathogenic *Vibrio* strains adhere to Gilt-head Sea bream (*Sparus aurata*) skin mucus (25), and *Vibrio harveyi* binds host GM4 ganglioside (NeuAcα2,3Galβ1Cer) (26, 27). However, very little is known about how fish pathogens interact with glycans, even though such knowledge has important implications for fish disease management.

The skin mucin glycan repertoire from Swedish and Norwegian Atlantic salmon were more closely related to each other than to the Tasmanian population, regardless of size and salinity. The Tasmanian Atlantic salmon originates from Nova Scotia in Canada, founder stocks were imported to New South Wales in Australia during the early 1960s, and from there to Tasmania in 1984. Fish belonging to the two metapopulations investigated by the present study show differences in chromosome number and structure, which allow them to be classified as having either a European or North American karyotype (19). European Atlantic salmon generally have 29 pairs of chromosomes and 74 chromosome arms (20), whereas it has been reported that North American Atlantic salmon have 27 chromosome pairs and 72 chromosome arms (21).

The Norwegian Atlantic salmon skin mucin *O*-glycans profile was relatively similar to the previously characterized Swedish population (13). A notable exception was the increase in expression of sialylated core 1 structures NeuAcα2–3Galβ1–3GalNAc and NeuAcα2–3Galβ1–3(NeuAcα2–6)GalNAc. However, the pattern of skin *O*-glycan expression in the Tasmanian population differed markedly from the Scandinavian samples; larger structures were more abundant, Kdn expression was absent or very low, and NeuGc expression was rare. The Tasmanian gastrointestinal mucins from both pyloric caeca and distal intestine had a notably higher abundance of fucosylated *O*-glycans compared with the Swedish samples. The majority of these fucoses were found linked to HexNAcs at the terminal end. The Tasmanian NeuAc containing structures were decreased compared with the Swedish salmon mucins in the distal intestine, and the small amount of NeuGc structures found was lower. In addition, some sulfated structures of low abundance were found in the pyloric caeca and distal intestine of Tasmanian samples which, to the best of our knowledge, is the first report of sulfated *O*-glycans in Atlantic salmon. Sulfated glycans have been reported for other fish species such as chub (*Squalius cephalus*), grass carp (*Ctenopharyngodon idella*), common carp (*Cyprinus carpio*) (28), Japanese butterfly ray (*Gymnura japonica*) and pond loach (*Misgurnus anguillicaudatus*) (29). A note should also be taken that the relative abundance of different glycan species may not necessarily reflect the actual ratio, because glycan species have different features when ionized, leaving some structures overrepresented and others underrepresented (30). However, because this study focuses on comparing the abundance of the same glycan between groups this inaccuracy is minimized, though the ratios of *e.g.* sialic acid-containing glycans is likely overestimated.

A hierarchical clustering of glycan abundances showed that skin mucin *O*-glycans are different from the *O*-glycans expressed in the pyloric caeca and distal intestine (data not shown). The skin mucins from Swedish and Norwegian salmon were closer related compared with the Tasmanian salmon, even though the Norwegian salmon were grown in seawater compared with freshwater for the other two geographical locations. Histochemical studies of gills have suggested that Atlantic salmon, brown trout and rainbow trout express more acidic mucins when grown in seawater (31), but no significant differences in relative abundance of skin mucin total sialylated structures were detected in the present study. All Swedish and Tasmanian samples clustered into sub-groups based on geographical origin suggesting region-specific differences. The results comparing Swedish Atlantic salmon three years apart as well as Tasmanian fish of various sizes subjected to different growth conditions suggests that temporal, age-related and environmental factors do have to the potential to affect glycosylation, albeit mildy. However, the differences between the Tasmanian and Swedish populations, and across epithelial locations, suggests geographic/genetic and tissue origin determine glycosylation to a much larger extent.

Our previous characterization of the Atlantic salmon *O*-glycome from a single location identified that interindividual variation was quite low (13). Because interindividual variation is considered a population-based defense, preventing the entire population from being wiped out by a single infection, these results may indicate a level of vulnerability in the face of disease threats. The current expansion of the Atlantic salmon *O*-glycome with another five cohorts confirmed low interindividual variation within each cohort, and even more so among Tasmanian than Scandinavian fish. The Swedish Atlantic salmon *O*-glycan interindividual variation was higher, although the total number of skin mucin glycans were greater in the Tasmanian cohort. One plausible explanation for this could be that the Swedish fish are from a wild population whereas the Norwegian and Tasmanian strains have been subjected to selective breeding. Within Tasmanian cohort 1 and Swedish cohort 4 from 2012, 71–83% and 51–57% of structures, respectively, were common to all fish. In a dataset of intestinal mucins from five healthy pigs the corresponding number was 39% (32), and in gastric mucin glycans from 10 human patients with varying health status ∼5% of structures were common (15). The relative abundance of these common structures in the fish constituted on average 94% of the total *O*-glycans in skin, 97% in pyloric caeca and 99% in distal intestine for the Tasmanian cohort 1. The lower percentage for skin mucins is because of one of the fish expressing NeuGc. The corresponding values in the Swedish cohort 4 were 97% in skin, 90% in pyloric caeca and 95% in distal intestine. These results indicate a low diversity of expressed mucin *O*-glycans within these populations. From comparing distributions of Spearman rank correlations within the affected populations, we found that all epithelial sites displayed mucin glycosylation of higher diversity in the Swedish Atlantic salmon compared with the Tasmanian cohort. This suggests that the Tasmanian diversity of glycan expression is lower than that of the Swedish in both the skin and the gastrointestinal tract.

In conclusion, the present study determined that within a geographical location, the Atlantic salmon *O*-glycan repertoire is relatively stable over time but displays some differences because of fish size and thus life stage. Clear differences were identified between geographical regions, with Atlantic salmon originating from Norway and Sweden exhibiting more similar glycan repertoires than those originating from Australia. However, the most abundant structures were often expressed in all populations, such as sialyl-Tn in skin and GalNAcα1–3(NeuAcα1–6)GalNAc in the gastrointestinal tract. Furthermore, although Atlantic salmon *O*-glycan repertoire from each region differed, the interindividual variation within each geographical region was consistently low. The large structural library compiled during this study identified possible glyco-determinants that could be used to investigate bacterial binding or used as artificial adhesion decoys for intervention therapies. It remains unknown whether the differences between populations observed during the present study are because of genetics, milieu or both, but either possibility presents opportunities to exploit. If genetic, populations can be selectively bred or moved for use in aquaculture to optimize resistance to specific diseases, whereas if because of local environment fish could potentially be induced to express *O*-glycans yielding the greatest benefit to aquaculture.

## DATA AVAILABILITY

MS/MS data on the tentative structures are available at http://unicarb-dr.biomedicine.gu.se/references/361. Raw data files are available at https://glycopost.glycosmos.org/entry/GPST000009.

## Supplementary Material

supplemental Table S1

Supplementary table 1

Supplementary figures

## Footnotes

^1^ The abbreviations used are:

GlcNAc: *N*-Acetyl glucoseamine
GalNAc: *N*-Acetyl galactoseamine
Glc: Glucose
Gal: Galactose
GalNAcol: N-Acetyl galactoseaminitol
Hex: Hexose
HexNAc: *N*-Acetyl hexoseamine
NeuAc: *N*-Acetylneuraminic acid
NeuGc: *N*-glycolylneuraminic acid
Kdn: Ketodeoxynonomic acid
LC-MS: Liquid chromatography-mass spectrometry
MS/MS: tandem mass spectrometry
*m/z*: mass-to-charge ratio.
