# Supplementary material for: Effects of Size and Geographical Origin on Atlantic salmon, Salmo salar, Mucin O-Glycan Repertoire
Source: Mol Cell Proteomics. 2019 Mar 28;18(6):1183–96. doi: 10.1074/mcp.RA119.001319 (PMC6553937; doi:10.1074/mcp.RA119.001319)
Supplement: supplemental Table S1 [file RA119.001319_index.html]

Supplement to Effects of size and geographical origin on Atlantic salmon, Salmo salar, mucin O-glycan repertoire | Molecular & Cellular Proteomics

## Supplemental Data

- Supplementary table 1 - Supplementary table of mass spectometry data results
- Supplementary figures - Supplementary Figure 1-4
