# Supplementary material for: Effects of Size and Geographical Origin on Atlantic salmon, Salmo salar, Mucin O-Glycan Repertoire
Source: Mol Cell Proteomics. 2019 Mar 28;18(6):1183–96. doi: 10.1074/mcp.RA119.001319 (PMC6553937; doi:10.1074/mcp.RA119.001319)
Supplement: Supplementary figures [file 142931_2_supp_306806_pp11z5.docx]

Supplementary figures

**Supplementary figure 1. Comparison of *O-*glycan profiles obtained from mucins isolated by crude extraction versus density gradient centrifugation.** A and B represents proximal intestinal mucus sample of two individual fish. Black bars represents the glycan profile obtained by LC-MS from mucins by the crude extraction method while the white bars signifies mucins purified by the density gradient centrifugation method.

**
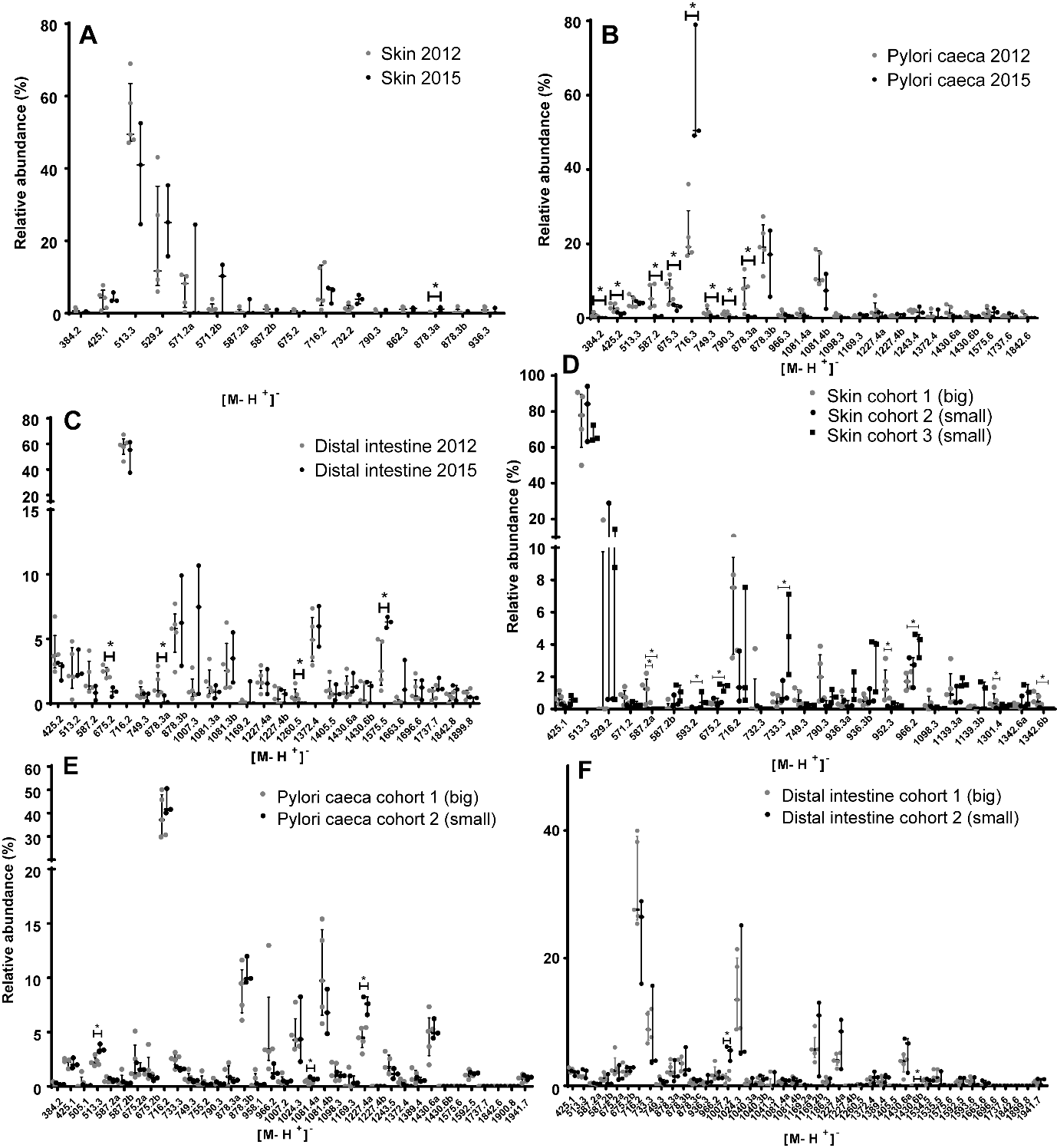
Supplemetary figure 2.** **A-C)** Comparison between *O*-glycan structures from samples taken from fish in 2012 and 2015. **D-F)** Comparison between *O*-glycan structures from big (20 month) and small (9 month) cohorts. Differences calculated using Mann-Whitney U-test and Kruskal-Wallis with Dunns´s multiple comparison.


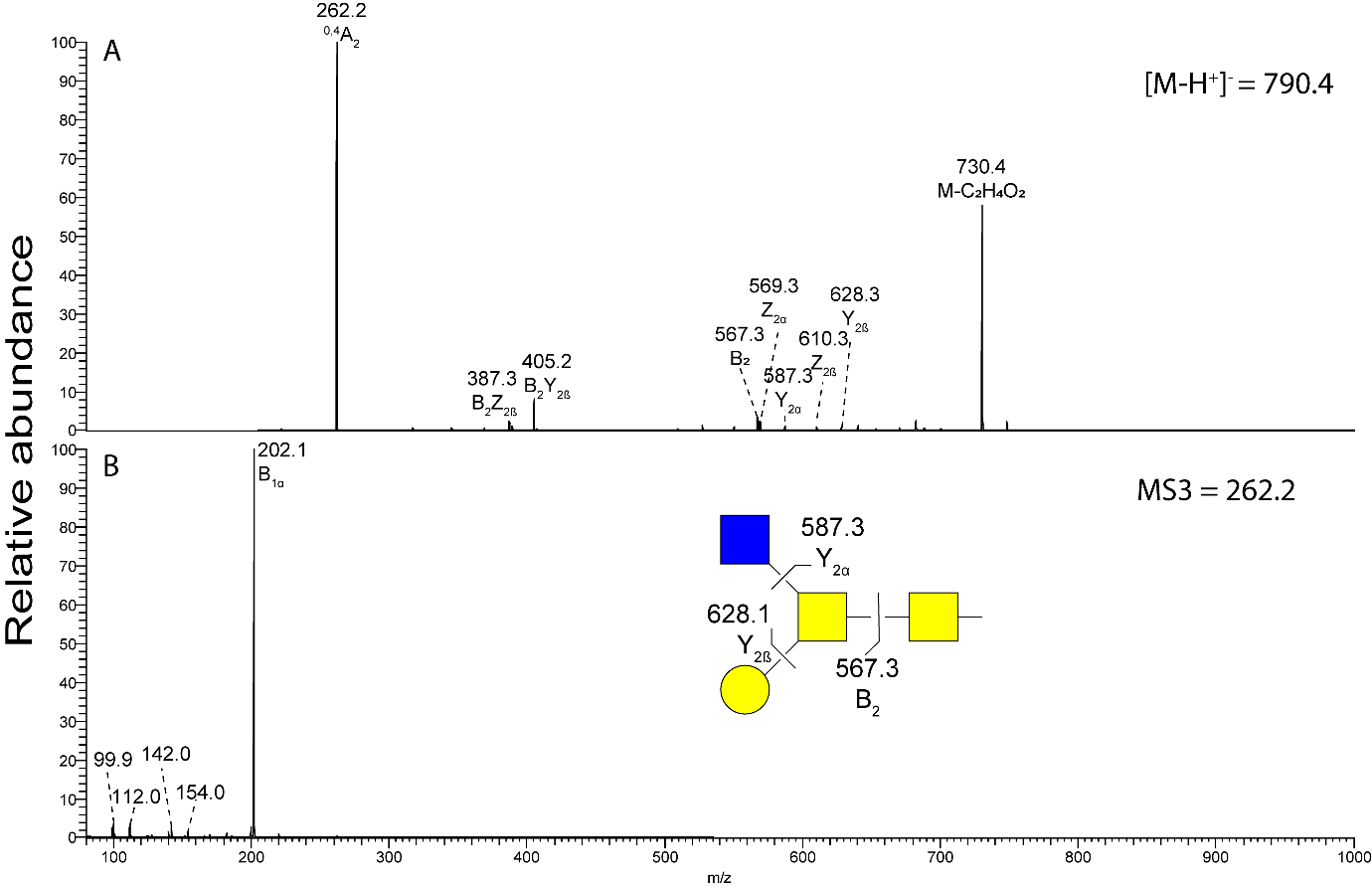


**Supplementrary figure 3. Determination of branched HexNAc in *m/z* 790 structure. A)** MS/MS of *m/z* 790.4 from Tasmanian skin mucins. **B)** MS^3^ of *m/z* 262.2 fragment of the *m/z* 790 parent ion. Fragments at *m/z* 100, 112, 142 and 154 assigns this as a ß linked GlcNAc according to Fang TT *et al.* (1).


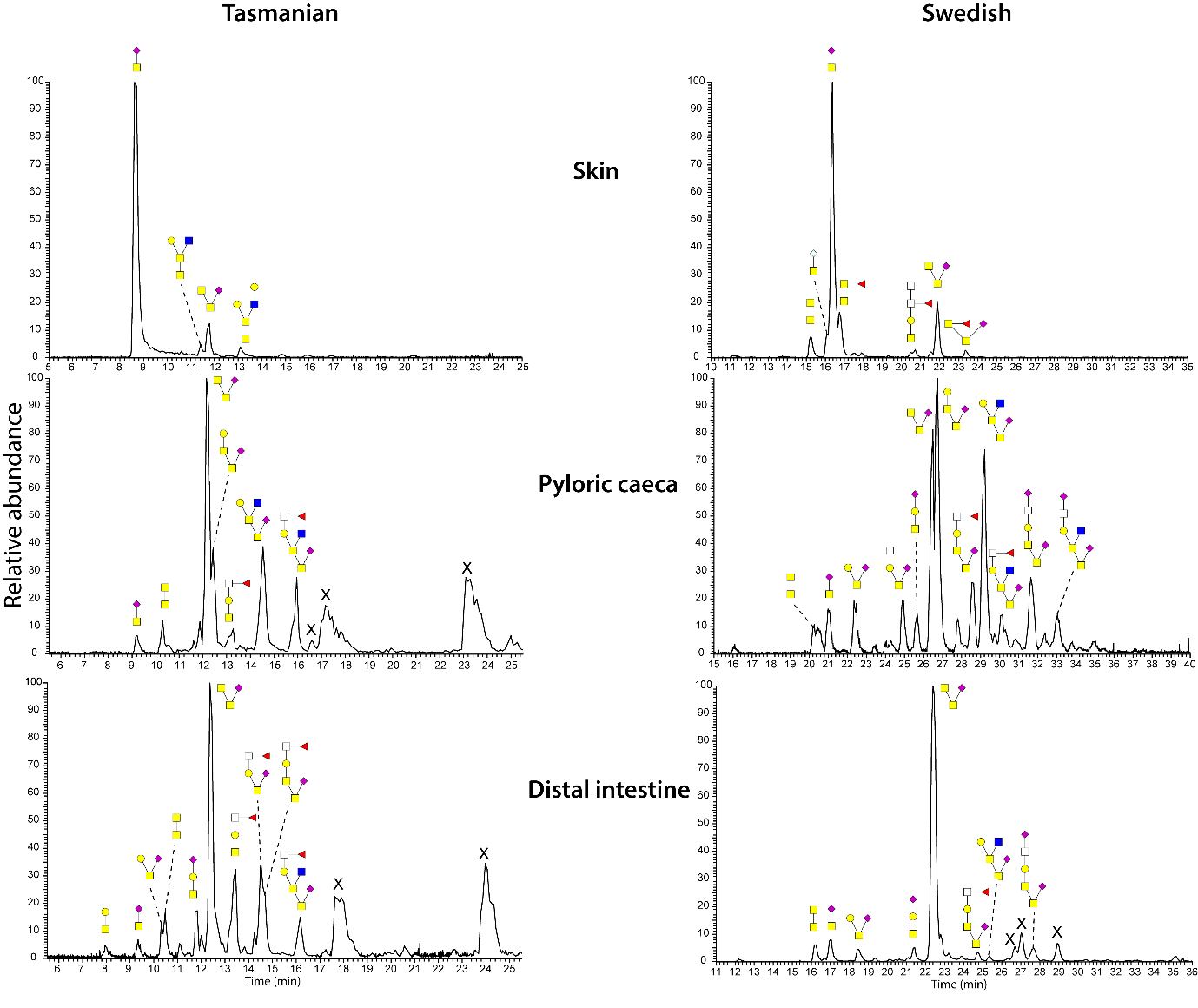


**Supplementrary figure 4. Examples of LC-MS chromatograms from Tasmanian and Swedish mucin *O*-glycans. Left)** Tasmanian samples were run on a 10 cm × 250 μm i.d PGC column packed in-house. **Right)** Swedish samples were run on a 15 cm × 250 μm i.d PGC column packed in-house. Due to wear on the PGC column, a gradual drift of retention times cause differences in elution time between the samples. Linkages of structures can be found in Supplementary Table 1. X in the annotations signify non-glycan peaks and adducts.

1. Fang, T. T., and Bendiak, B. (2007) The Stereochemical Dependence of Unimolecular Dissociation of Monosaccharide-Glycolaldehyde Anions in the Gas Phase:  A Basis for Assignment of the Stereochemistry and Anomeric Configuration of Monosaccharides in Oligosaccharides by Mass Spectrometry via a Key Discriminatory Product Ion of Disaccharide Fragmentation, m/z 221. *Journal of the American Chemical Society* 129, 9721-9736
